# Diagnosis of ischemic optic neuropathy caused by dissection of the internal carotid artery: A case report

**DOI:** 10.1097/MD.0000000000020034

**Published:** 2020-08-14

**Authors:** Xiangrong Zheng, Yunpeng Wang, Guocang Chen, Chuanyong Ma, Weiming Yan, Meizhu Chen

**Affiliations:** Department of Ophthalmology, The 900th Hospital of Joint Logistic Support Force, PLA (Clinical Medical College of Fujian Medical University, Dongfang Hospital Affiliated to Xiamen University), Fuzhou, China.

**Keywords:** case report, dissection, internal carotid artery, ischemic optic neuropathy, visual-evoked potential

## Abstract

**Rationale:**

Ischemic optic neuropathy (ION), due to diseases of the arteries supplying the optic nerve, is an ischemic damage of the optic nerve. This report highlights a case with monocular decreasing visual acuity caused by dissection of the internal carotid artery (ICA), which is a relatively rare cause for ION.

**Patient concerns:**

A 44-year-old woman presented with a decreasing visual acuity and defected visual field in the right eye for 1 week. The best corrected visual acuity (BCVA) was 20/400 in the right eye, and 20/20 in the left eye. In the right eye, the pupil showed little reaction to light with a relative afferent pupillary defect. The visual field test disclosed a defect in the inferior field connecting to the blind spot. Electroretinogram recording showed no obviously declined retinal function. No recognizable waveforms were presented in pattern visual–evoked potential (PVEP) examination, whereas the flash visual–evoked potential result revealed a delayed peak time and a reduced amplitude of P2-wave.

**Diagnosis:**

The patient was diagnosed as ION with the aid of computed tomographic angiography of the brain and neck, which revealed a stenosis in the right ICA and an occlusion in the right cerebral middle artery. The stenosis was verified as dissection of the ICA by digital subtraction angiography.

**Interventions:**

Based on the clinical findings, stent implantation inside the right ICA was performed.

**Outcomes:**

The ICA was recanalized soon and the BCVA of the right eye was improved to be 20/25 five months later. A second PVEP examination revealed a recognizable waveform in the right eye, although the peak time and amplitude of the P100-wave was a bit abnormal compared to that of the left eye.

**Lessons:**

ION with the sign of decreasing monocular visual acuity could occur due to dissection of the ICA, with no obvious neurologic symptom at the beginning. The present case emphasizes the importance of suspicion of ICA problems as the underlying cause for ION, which could help to take in-time measure to save the vision and avoid further complications.

## 1 Introduction

Ischemic optic neuropathy (ION) is the disorder of optic nerve due to the decreased blood flow in the supplying arteries. Usually, ION could be divided into the subtypes of anterior ION (AION) and posterior ION (PION), according to the involved area of lesion.^[1]^ The characterized and common symptoms of ION include a monocular painless decreasing vision, a relative afferent pupillary defect (RAPD), and the altitudinal, especially inferior, visual field defects. The hyperemic swelling of the optic disc is the typically clinical sign of AION, whereas the optic disc remains normal in PION. However, the pale optic disc would come into appearance about 1 month after the vision loss in PION.^[2]^

Diagnosis of ION usually relies on the confirmation of occlusion or stenosis of the supplying arteries of optic nerve in addition to the clinical symptom and signs. As the symptom of ION resembles closely to that of ophthalmo neuromyelitis, it is not easy to distinguish between these two diseases in some cases.^[3]^ Furthermore, there are several arteries supplying the optic nerve. Anatomically, the central retinal artery and posterior ciliary artery supply the anterior portion of the optic nerve, whereas the multiple pial vessels arising from the ophthalmic artery supplies the posterior portion of the optic nerve.^[4]^ Sometimes, it is difficult to figure out the underlying problem of the affected supplying arteries. Therefore, the ION might be delayed in diagnosis clinically.

Internal carotid artery (ICA) comprises the supplying arteries for optic nerve, especially for the posterior part. Occlusion or stenosis of ICA would lead to reduced blood supply for optic nerve.^[5]^ In greater detail, the ICA dissection is one of the main reasons for the ICA occlusion or stenosis. So far, ION related to ICA dissection has not been largely reported.^[6]^ We here reported a patient with ION due to ICA dissection and discussed previously reported cases, focusing on the process of the diagnosis of ION.

## 2 Case report

A 44-year-old woman was referred to our department complained of a decreasing visual acuity and loss of the inferior visual field in the right eye. She had no nausea or headache. No other physical and neurological symptoms were positive. Her family history was negative for ocular diseases. She gave the informed consent for publication of the case.

**Figure 1:**
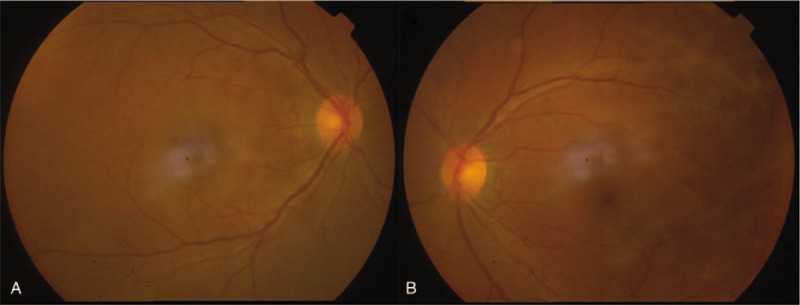
Fundus photograph of both eyes (A, the right eye, Oculus Dexter (OD); B, the left eye, Oculus Sinister (OS)). No obvious abnormalities were present in both eyes.

On admission, ophthalmic examinations showed the best corrected visual acuity (BCVA) was 20/400 in the right eye, and 20/20 in the left eye. The diameter of the right pupil was 4 mm, which was a bit larger than the left. In addition, the right pupil only minimally reacted to direct or consensual light stimulation, accompanied with an RAPD. Funduscopic examination of both eyes revealed no obvious abnormality of the optic disc, retina, and retinal vessels (Fig. 1).

**Figure 2:**
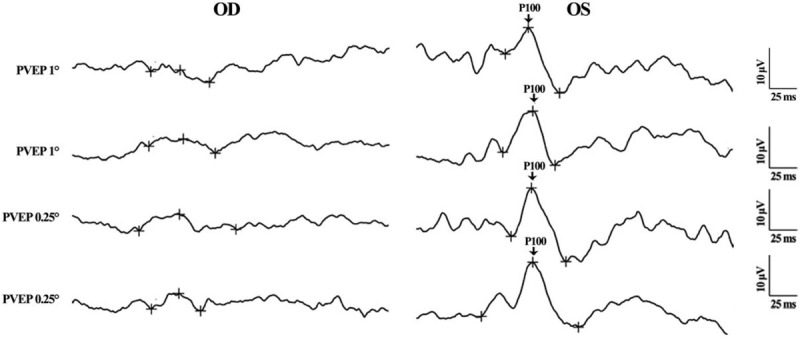
Pattern visual–evoked potential (PVEP) of both eyes (Oculus Dexter (OD): the right eye; Oculus Sinister (OS): the left eye). No recognizable waveform in the right eye was found. P100: the P100-wave of the PVEP examination.

**Figure 3:**
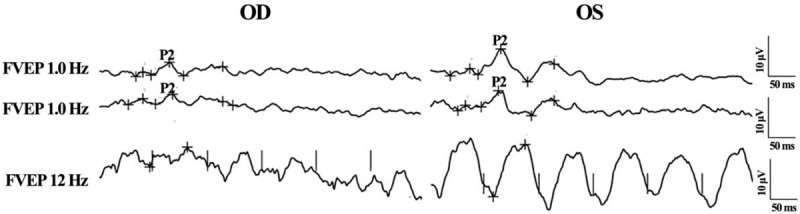
Flash visual–evoked potential (FVEP) of both eyes (Oculus Dexter (OD): the right eye; Oculus Sinister (OS): the left eye). A reduced amplitude and a delayed phase of P2-wave were found in OD. P2: the P2-wave of the FVEP examination.

**Figure 4:**
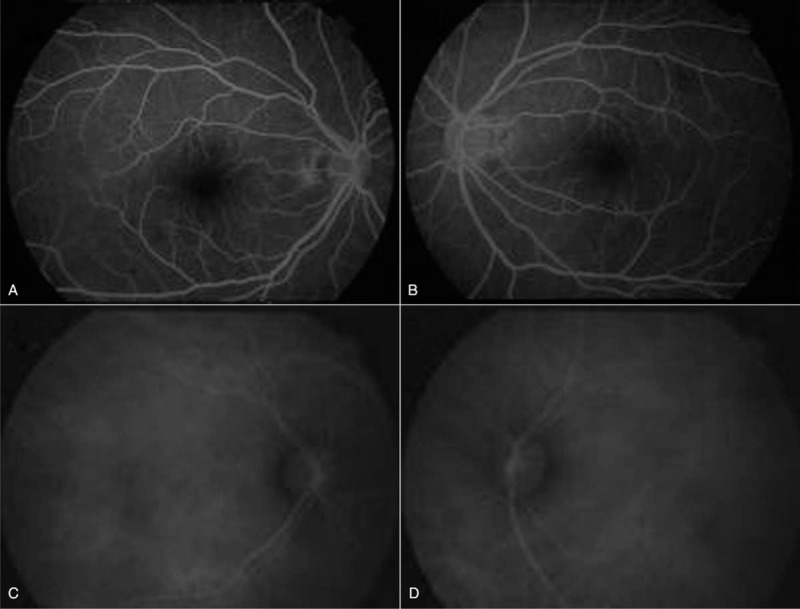
Fundus fluorescence angiography (FFA) and indocyanine green angiography (ICGA) of both eyes (A, the right eye, OD of FFA; B, the left eye, OS of FFA; C, the right eye, Oculus Dexter (OD) of ICGA; D, the left eye, Oculus Sinister (OS) of ICGA). The FFA and ICGA found no leakage in both eyes throughout all the phrases.

**Figure 5:**
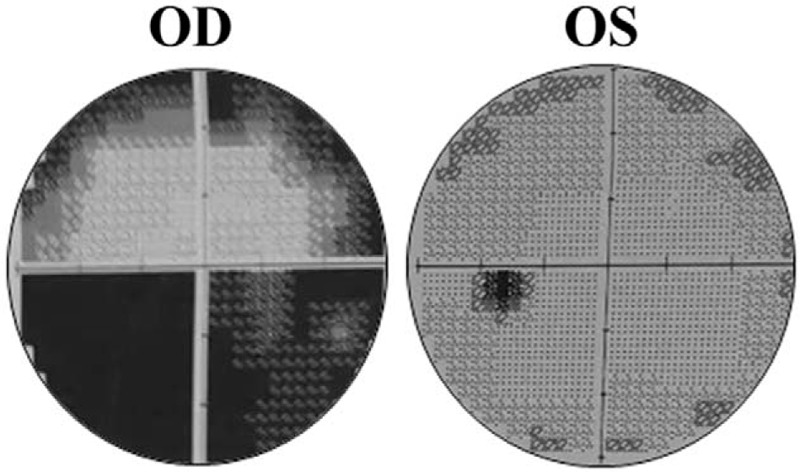
Visual field test of both eyes (Oculus Dexter (OD): the right eye; Oculus Sinister (OS): the left eye). The visual field test of the right eye disclosed a defect in the whole inferior field connecting to the blind spot.

The B-scan ultrasonography found no obvious abnormality in both eyes. The retinal and choroid structure in the right and left eyes were normal under optical coherent tomography (OCT) examination. There were, however, not recognizable waveforms in the right eye by pattern visual–evoked potential (PVEP) examination. The results of flash visual–evoked potential revealed a delayed of peak time and a reduced amplitude of P2-wave in the right eye compared to that of the left eye (Figs. 2 and 3). The electroretinogram (ERG) recording showed no obvious change of the retinal function in both eyes. The fundus fluorescence angiography and indocyanine green angiography did not reveal any leakage of fluorescence or indocyanine throughout all the phases for both eyes (Fig. 4). The visual field test disclosed a defect in the inferior field connecting to the blind spot of the right eye (Fig. 5).

**Figure 6:**
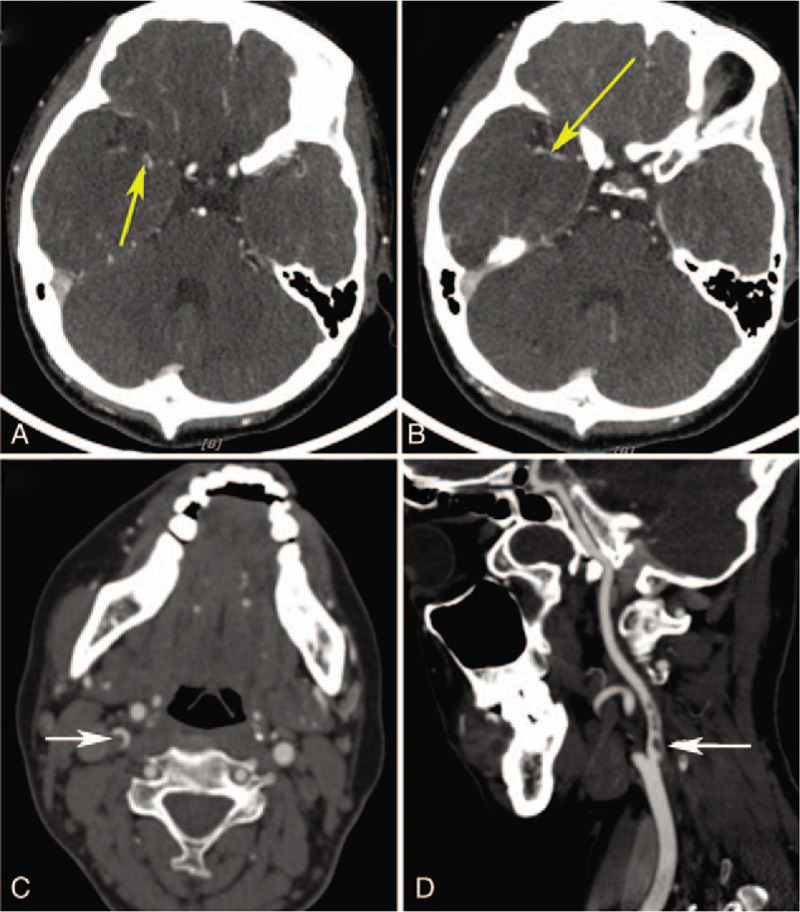
Computed tomographic angiography (CTA) of the brain and neck (A, B: the cerebral angiography; C, D: the cervical angiography). The CTA revealed an occlusion of the distal segment in the right cerebral middle artery (yellow arrow) and a 21.5% stenosis of the right internal carotid artery (ICA) (white arrow).

**Figure 7:**
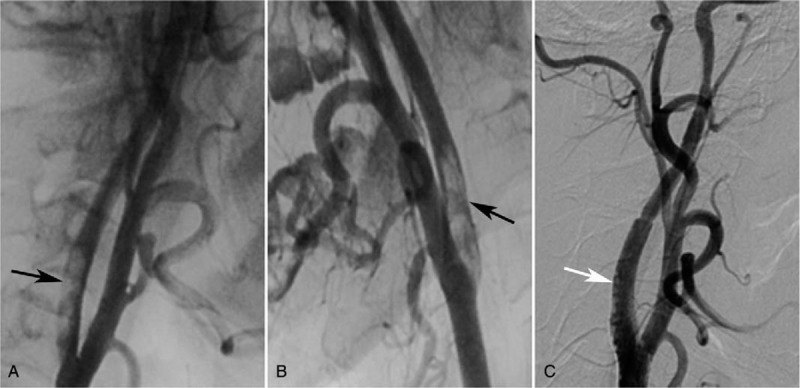
Digital subtraction angiography (DSA) examination of the cervical vessels before (A, B) and after (C) stent implantation in the right internal carotid artery (ICA). The DSA confirmed the severe dissection of right ICA, which contributed to the almost 90% stenosis (black arrow in A and B). After the stent implantation, the canal of right ICA was opened again (white arrow in C).

**Figure 8:**
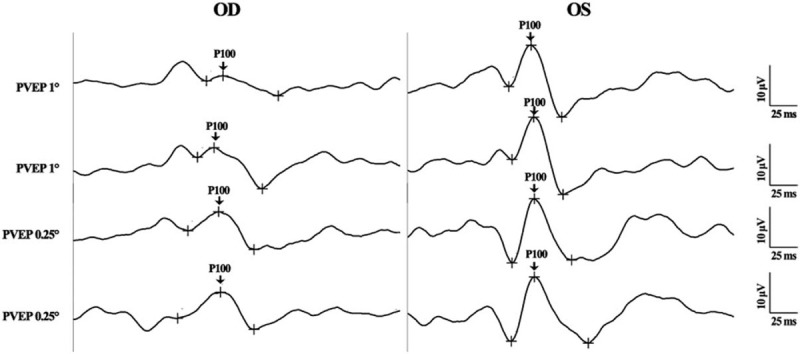
Pattern visual–evoked potential (PVEP) of both eyes 5 months after stent implantation in the right internal carotid artery (ICA). (Oculus Dexter (OD): the right eye; Oculus Sinister (OS): the left eye). A recognizable waveform of PVEP was presented in the right eye, even though with a reduced amplitude and a delayed phase of P100-wave compared to that of the left eye. P100: the P100-wave of the PVEP examination.

A computed tomographic angiography (CTA) of the brain and neck did not reveal any problems among the supplying vessels, including the ICA. The patient, however, developed an incontinence 5 days later, which was deemed to be caused by the cerebral infarction. She was referred to the Neurology Department immediately and a second CTA of the brain and neck was performed. The result disclosed an occlusion in the right cerebral middle artery and a 21.5% stenosis of the right ICA and (Fig. 6). The later digital subtraction angiography revealed that the dissection of ICA was the underlying reason for the stenosis, which resulted in an almost 90% stenosis of the right ICA at the time of examination. This patient later received the stent implantation insides the right ICA (Fig. 7). The ICA was recanalized soon and the BCVA in the right eye was improved to be 20/25 five months after the surgery. PVEP was also performed then and the results revealed a recognizable waveform in the right eye, with a reduced amplitude and a delayed peak time of P100-wave compared to the left eye (Fig. 8).

## 3 Discussion

Here we presented a case of ION with cerebral infarction due to the stenosis in the right ICA caused by dissection. Our patient had the symptom of the decreased visual acuity and quadrant visual field defect, which were typical in the ION due to the ischemia setting of optic nerve.^[7]^

ION is commonly defined as an inadequate blood supply to the optic nerve in the absence of retinal and vascular abnormalities on fundus examination. Factors that affect the ocular blood perfusion to the supplying vessels of optic nerve would lead to the decreased blood flow and possibly result in ION.^[8]^ Inflammatory of the blood vessels has been reported to the cause arteritic ION, with the giant cell arteritis being the most ominous.^[9,10]^ Other factors, such as enhanced intraocular pressure, blood hypercoagulable state, and hypotension have also been related to the ION, which belong to the nonarteritic ION.^[11]^ The underlying mechanism of ION involves the failure of cellular energy due to the insufficient blood flow.^[12]^

Generally, ION could be categorized into the AION and PION based on the affected area of the optic nerve. Actually, there is a complex vascular supply to the optic nerve through several vessels and most of these supplying vessels come from the ICA.^[13]^ Stenosis and/or occlusion of the ICA would reduce ocular blood supply and result as a contributor to ION. Newman^[14]^ presented one case of ION caused by occlusion in the ophthalmic artery, which is a branch of ICA. In particular, ION due to ICA dissection, which is recognized as a major cause of stenosis or occlusion, has been reported only in a few cases. Specifically, McNeill et al^[15]^ described a 48-year-old woman who had the left ICA dissection and suffered monocular visual blurring, with the signs of a temporal disc margin on funduscopy. Other literatures reported large series of ICA dissection but not mentioned the ophthalmic details. In our case, the ION symptom and sign were obviously presented, such as the painlessly decreased visual acuity, the defected visual field, and the affected visual evoked potentail (VEP). In addition, we detected a positive RAPD. The RAPD during ION has also been observed, which is suggested to reflect the ischemic state of the ciliary ganglion and a relative hypoperfusion of the ocular tissues.^[16]^

In our case, the cerebral infarction led us to the disclosure of ICA stenosis which was due to the dissection. As the ICA also supplies the cerebral tissues through the cerebral middle artery, there is no doubt that the stenosis of ICA could also cause the cerebral infarction.^[17]^ Thus, neurologic symptoms, such as incontinence, happened in our patient. In great detail, the clinical sign of ION existed long before the cerebral infarction in our case. Similarly, other reports also found that the ION symptoms appeared before the infarction. Rivkin et al^[18]^ reported a case who presented with PION followed by a massive cerebral infarct in two days. The reason might be ascribed to the self-adjusted abilities of the cerebral vessels, which could last longer than other tissues in case of ischemia.

Admittedly, we did not conclude the ION as the AION or PION in our case. Instead, the diagnosis might be the combination of both. As there was a lack of the swelling in the optic disc, the arteries supplying the anterior part of optic nerve were deemed to be less affected. Another explanation for the spare of the AION might be the higher self-adjusted abilities of the anterior arteries compared to the posterior parts, although the anterior and posterior arteries all come originally from the ICA.^[19]^

What's lucky in our case was the great recovery of visual acuity and the PVEP waveform after the stent surgery, even though the optic nerve had been experienced a low perfusion for days. As no obvious swelling of the optic disc was found, the period of the ION might not be too long at the time of admission. This reason might account for the recovery of the visual acuity. ION, however, caused by the ICA dissection reported in other cases did not get a satisfactory recovery of visual acuity, which might be due to the long period of ischemia.^[20]^

In our case, no obvious abnormalities of retinal structure and function were found through OCT and ERG examinations (data not shown), indicating the relative spare of retina. Similarly, not any visible retinal emboli in ION cases was revealed in other reports.^[21]^ In addition, ischemic retinopathy or any signs suggestive of central retinal artery occlusion, which is a hallmark of chronic hypoperfusion of the eye, was not found during ION, either.^[22]^ The possible reason for the relative retinal sparing might be ascribed to the preserved blood supply to retina through the choroidal anastomosis during the early phase of ICA and the relatively rapid recanalization of the ICA in some cases.

Intriguingly, the underlying reason for ICA stenosis in our case was not figured out through the first CTA examination. As the decreased visual acuity persisted, we could not explain it with other arteries abnormalities or the optic neuritis. The second CTA after the appearance of incontinence disclosed the ICA lesions. Probably, the dissection of ICA gradually happened and formed during the time between the two CTA examinations. Maybe the lesion of ICA was too tiny to be found before the real dissection occurred.^[23,24]^

## 4 Conclusion

**W**e presented a case of ION in which the ICA dissection turned out to be the underlying reason. Threshold of suspicion of the ICA problems during ION should be lowered, and proper treatment should be initiated at time to avoid further complications, such as cerebral infarction, and to assure a good recovery of visual acuity.

## Author contributions

**Conceptualization:** Yunpeng Wang, Xiangrong Zheng, Weiming Yan, Meizhu Chen, Guocang Chen.

**Data curation:** Xiangrong Zheng, Weiming Yan, Guocang Chen.

**Supervision:** Weiming Yan, Meizhu Chen.

**Writing** – **original draft:** Weiming Yan, Yunpeng Wang, Xiangrong Zheng, Guocang Chen.

**Writing – review and editing**: Weiming Yan, Meizhu Chen.

## References

[1] Albarrak AM Mohammad Y Hussain S Simultaneous bilateral posterior ischemic optic neuropathy secondary to giant cell arteritis: a case presentation and review of the literature. BMC Ophthalmol 2018;18:317. 30541489 10.1186/s12886-018-0994-9 PMC6292061

[2] Fard MA Yadegari S Ghahvechian H Optical coherence tomography angiography of a pale optic disc in demyelinating optic neuritis and ischemic optic neuropathy. J Neuroophthalmol 2019;39:339–44. 30893268 10.1097/WNO.0000000000000775

[3] Xie Y Yang X Zhang W A comparative study on clinical features of anterior ischemic optic neuropathy and idiopathic optic neuritis. Minerva Med 2019;110:475–7. 30784250 10.23736/S0026-4806.19.05976-7

[4] Oh DJ Chhadva P Kanu LN Sudden-onset blindness from a spontaneous carotid-cavernous fistula with secondary central retinal artery occlusion and posterior ischemic optic neuropathy. Neuroophthalmology 2019;43:107–13. 31312235 10.1080/01658107.2018.1488979 PMC6619923

[5] Garcia-Medina JJ Del-Rio-Vellosillo M Fares-Valdivia J Optic nerve hypoplasia and internal carotid artery hypoplasia: a new association. Can J Ophthalmol 2017;52:e173–7. 28985826 10.1016/j.jcjo.2017.05.006

[6] Song JX Lin XM Hao ZQ Ocular manifestations of internal carotid artery dissection. Int J Ophthalmol 2019;12:834–9. 31131245 10.18240/ijo.2019.05.21 PMC6520286

[7] Han S Jung JJ Kim US Differences between non-arteritic anterior ischemic optic neuropathy and open angle glaucoma with altitudinal visual field defect. Korean J Ophthalmol 2015;29:418–23. 26635459 10.3341/kjo.2015.29.6.418 PMC4668258

[8] Harvey JP Effect of intravenous anesthetic agents on ocular nerve blood supply and their role in nonarteritic ischemic optic neuropathy. J Neuroophthalmol 2019;[Epub ahead of print].

[9] Dogra M Singh R Dogra MR Giant cell arteritis related arteritic anterior ischemic optic neuropathy: clinico-pathological correlation. Indian J Ophthalmol 2019;67:142. 30574925 10.4103/ijo.IJO_881_18 PMC6324127

[10] Lee EJ Woo KA Koo DL Giant cell arteritis associated arteritic anterior ischemic optic neuropathy: sudden vision loss on the contralateral side of headache. J Clin Neurol 2018;14:577–9. 30198237 10.3988/jcn.2018.14.4.577 PMC6172488

[11] Weiss JN Levy S Benes SC Stem Cell Ophthalmology Treatment Study: bone marrow derived stem cells in the treatment of non-arteritic ischemic optic neuropathy (NAION). Stem Cell Investig 2017;4:94. 10.21037/sci.2017.11.05 PMC5723737 29270420

[12] Khalilpour S Latifi S Behnammanesh G Ischemic optic neuropathy as a model of neurodegenerative disorder: a review of pathogenic mechanism of axonal degeneration and the role of neuroprotection. J Neurol Sci 2017;375:430–41. 28320183 10.1016/j.jns.2016.12.044

[13] Machalinska A Kowalska-Budek A Kawa MP Association between asymptomatic unilateral internal carotid artery stenosis and electrophysiological function of the retina and optic nerve. J Ophthalmol 2017;2017:4089262. 28491467 10.1155/2017/4089262 PMC5405584

[14] Newman NJ Perioperative visual loss after nonocular surgeries. Am J Ophthalmol 2008;145:604–10. 18358851 10.1016/j.ajo.2007.09.016 PMC2989384

[15] McNeill DJ Dreisbach J Marsden RJ Spontaneous dissection of the internal carotid artery. Its conservative management with heparin sodium. Arch Neurol 1980;37:54–5. 7350903 10.1001/archneur.1980.00500500084015

[16] Kernstock C Beisse F Wiethoff S Assessment of functional and morphometric endpoints in patients with non-arteritic anterior ischemic optic neuropathy (NAION). Graefes Arch Clin Exp Ophthalmol 2014;252:515–21. 24477537 10.1007/s00417-014-2572-z

[17] Sahan MH Asal N Bayar MN Critical stenosis of the internal carotid artery: variability in vertebral artery diameters and areas of cerebral chronic infarction in computed tomography. J Craniofac Surg 2019;30:e388–92. 31299787 10.1097/SCS.0000000000005225

[18] Rivkin MJ Hedges TR Logigian EL Carotid dissection presenting as posterior ischemic optic neuropathy. Neurology 1990;40:1469. 10.1212/wnl.40.9.1469 2392239

[19] Patel HR Margo CE Pathology of ischemic optic neuropathy. Arch Pathol Lab Med 2017;141:162–6. 28029908 10.5858/arpa.2016-0027-RS

[20] Li A Li L Li M A new characterization for nonarteritic anterior ischemic optic neuropathy. Int J Clin Exp Med 2015;8:18681–8. 26770482 PMC4694382

[21] Schmidt D Richter T Vvon Reutern GM Acute circulatory disorders of the eye. Clinical findings and results of Doppler sonography of the internal carotid artery. Fortschr Ophthalmol 1991;88:84–98. 2045032

[22] Hayreh SS Ocular vascular occlusive disorders: natural history of visual outcome. Prog Retin Eye Res 2014;41:1–25. 24769221 10.1016/j.preteyeres.2014.04.001 PMC4073304

[23] Kawabe K Nagaoka T Iguchi H Optic nerve MRI enhancement in posterior ischaemic optic neuropathy due to internal carotid artery dissection. Clin Neurol Neurosurg 2010;112:350–2. 20106590 10.1016/j.clineuro.2009.12.008

[24] Biousse V Touboul PJ D’Anglejan-Chatillon J Ophthalmologic manifestations of internal carotid artery dissection. Am J Ophthalmol 1998;126:565–77. 9780102 10.1016/s0002-9394(98)00136-6

